# The Prevalence and Risk Factors of Low Back Pain Among Office Workers in Saudi Arabia

**DOI:** 10.7759/cureus.44996

**Published:** 2023-09-10

**Authors:** Ibrahim A Bin Ahmed, Abdulmalik E Aldhafyan, Ahmed A Basendwah, Turki Y Alassaf, Hamad N Alhamlan, Abdullah H Alorainy, Abdalaziz B Alyousef

**Affiliations:** 1 Department of Family Medicine, Imam Mohammad Ibn Saud Islamic University, Riyadh, SAU; 2 College of Medicine, Imam Mohammad Ibn Saud Islamic University, Riyadh, SAU

**Keywords:** risk factor, prevalence, office workers, saudi arabia, low back pain

## Abstract

Background and objective

Low back pain (LBP) is a significant and prevalent musculoskeletal disorder associated with the workplace that impacts individuals, families, communities, healthcare systems, and companies. Although LBP prevalence and risk factors have been studied in various professional categories in Saudi Arabia, there is no data on the prevalence of LBP among office workers and related risk factors. This study aimed to determine the prevalence of LBP among office workers in Saudi Arabia and identify major risk factors.

Methodology

This cross-sectional study was conducted among office workers in Saudi Arabia of both genders aged over 18 years living in five geographical regions: Central, Western, Southern, Eastern, or Northern. A self-administered questionnaire was distributed among office workers using an online survey. The questionnaire comprised sociodemographic characteristics, the prevalence of work-related LBP, and work-related characteristics.

Results

Among 604 office workers, 51.7% were males and 43.5% were aged between 18 and 30 years old. The prevalence of work-related LBP in this study was 59.9%. Independent risk factors for LBP include being overweight or obese, sleep disturbance, previous history of back trauma, increasing years of working in the office, changes made to workstation/work habits to reduce the risk of LBP, and frequent work stress. Protective risk factors for LBP include regular physical exercise and satisfaction with the current job.

Conclusions

LBP was widely prevalent among office workers in Saudi Arabia. Office workers with elevated body mass index (BMI) who had a previous history of back trauma and had sleep disturbance or work stress in the past month were at significant risk for LBP. Occupational health and safety programs are vital for building ergonomically safe working conditions, and regular physical exercise promotion could alleviate the risk of LBP at work.

## Introduction

Low back pain (LBP) is considered a significant and one of the most prevalent musculoskeletal disorders associated with the workplace [1,2]. It is defined as "pain localized between the 12th rib and the inferior gluteal folds, with or without leg pain" [3]. Individuals and their families, communities, healthcare systems, and companies are greatly affected by LBP in multiple aspects [4]. According to recent studies, the number of people affected by LBP is expected to increase significantly over the next several years [5]. The number of people affected by LBP globally has been rising throughout the years, reaching 577 million, with a prevalence of 7.5% in 2017 [6]. A cross-sectional study conducted in Lebanon in 2015 showed that 44.8% of office workers were affected by back pain [2].

Multiple studies have been conducted to determine LBP's risk factors and prevalence [7,8,9]. Smoking, high body mass index (BMI) [10], high workload, frequent lifting, postural stress, and psychological distress are all risk factors that have been shown to increase the risk of developing LBP [11]. Another study found that increased age and sitting for long periods at work and in front of the computer increases the risk of developing LBP [12].

While several studies have investigated the prevalence and risk factors of LBP among different professional categories in Saudi Arabia [13,14,15], there is no data on the prevalence of LBP among office workers and its related risk factors. Therefore, the objectives of this study are as follows:

1. To determine the prevalence of LBP among office Workers in Saudi Arabia

2. To find out the major risk factors that lead to LBP among office workers in Saudi Arabia

## Materials and methods

Methodology

Study Design

This is a descriptive-analytical cross-sectional study conducted in the Kingdom of Saudi Arabia between April 2023 and August 2023. The inclusion criteria include office workers of both genders aged over 18 years living in the five geographical regions: central, western, southern, eastern, and northern. The exclusion criteria include being less than 18 years old, non-office workers, and office workers outside the Kingdom of Saudi Arabia. The study was approved by the research ethics committee at Imam Mohammad Ibn Saud Islamic University, Riyadh, Saudi Arabia (495/2023), and it followed the National Committee of Bioethics guidelines.

Sample Size

The study was conducted in the Kingdom of Saudi Arabia in five geographical regions: central, western, southern, eastern, and northern. The adult population of Saudi Arabia is approximately 23 million. The necessary sample size to reach a 95% confidence interval (CI) and a margin of error of 5% was 385 participants calculated by using the OpenEpi web tool. A total of 604 participants completed the questionnaire.

Application of the Questionnaire

The instrument used is an electronic questionnaire that was validated by experts in the field. Furthermore, a pilot study was also carried out on a small group of office workers to evaluate the clarity of the questionnaire. The pilot study participant's comments were used to further improve the questionnaire. The electronic questionnaire was created on Google Forms in Arabic and English and was distributed to the participants; it included questions about demographic characteristics (age, gender, height, weight, smoking habits, daily physical activity, sleep disturbance, and history of previous back trauma). Finally, we added questions to evaluate the occupational risk factors related to LBP.

Statistical Analysis

Frequency and proportion (%) were used to summarize all the data in this project. The relationship between the prevalence of LBP regarding the sociodemographic and clinical characteristics of office workers has been conducted using the chi-square test. Significant results were then tested in a multivariate regression model to determine the significant independent predictor of LBP with a corresponding odds ratio and a 95% CI. A *P*-value of 0.05 is taken as a cutoff point for statistical significance. All statistical data were computed using SPSS Version 26 (IBM Corp., Armonk, NY, USA).

## Results

Out of the 703 participants who completed the questionnaire, 604 were eligible for enrollment in the study as they met the inclusion criteria. A total of 99 participants were excluded from the study due to not meeting the specified criteria. As described in Table 1, 263 (43.5%) participants were aged between 18 and 30 years, with 312 (51.7%) being males. Two hundred and fifty (41.4%) participants were living in the central region. The proportion of smoking participants was 22.4% (135). 200 (33.1%) respondents were overweight. Only 82 (13.6%) participants exercised more than three days per week, and 87 (14.4%) had sleep disturbance on the same occasion. The prevalence of participants with a history of back trauma was (117, 19.4%).

**Table 1:** Sociodemographic characteristics of participants (n = 604).

Study variables	*n* (%)
Age group (years)	
18-30	263 (43.5)
31-40	191 (31.6)
>40	150 (24.8)
Gender	
Male	312 (51.7)
Female	292 (48.3)
Residence region	
Central region	250 (41.4)
Eastern region	98 (16.2)
Northern region	60 (09.9)
Western region	125 (20.7)
Southern region	71 (11.8)
Smoker	
Yes	135 (22.4)
No	469 (77.6)
BMI level	
Underweight (<18.5 kg/m^2^)	26 (04.3)
Normal (18-24.9 kg/m^2^)	278 (46)
Overweight (25-29.9 kg/m^2^)	200 (33.1)
Obese (≥30 kg/m^2^)	100 (16.6)
How many days do you exercise weekly?	
1 day	119 (19.7)
2 days	103 (17.1)
3 days	65 (10.8)
>3 days	82 (13.6)
Never	235 (38.9)
How many times a week do you have sleep disturbance?	
1 day	121 (20)
2 days	90 (14.9)
3 days	62 (10.3)
>3 days	87 (14.4)
Never	244 (40.4)
Previous history of back trauma	
Yes	117 (19.4)
No	487 (80.6)

**Figure 1:**
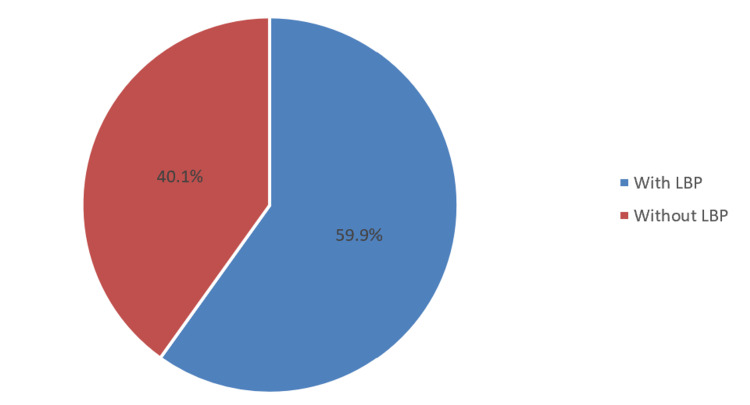
Prevalence of LBP among office workers. LBP, low back pain

In Table 2, the prevalence of office workers who experience LBP while working in the office was 59.9% (362; Figure 1), with 120 (19.9%) participants experiencing the pain at least weekly, wherein 160 (26.5%) reported 4 to 6 (out of 10) severity of pain. One hundred thirty-one (21.7%) respondents limited their activities due to LBP. Two hundred and fifty-six (42.4%) office workers worked in the office for at least 5 to 10 years, and 388 (64.2%) worked in the office desk sitting for at least eight hours per day. A great proportion of the office workers indicated having two days rest period. Interestingly, 281 (46.5%) of office workers changed their workstations/work habits to reduce the risk of LBP. The prevalence of office workers who were satisfied with their jobs was 78.1% (472). In addition, 228 (37.7%) experienced sometimes work stress during the last month.

**Table 2:** Prevalence of LBP and work-related characteristics of participants (n = 604). LBP, low back pain

Variables	*n* (%)
Have you ever experienced LBP while working in the office?	
Yes	362 (59.9)
No	242 (40.1)
How often do you experience LBP while working in the office?	
Daily	77 (12.7)
Weekly	120 (19.9)
Monthly	101 (16.7)
Rarely	64 (10.6)
Never	242 (40.1)
How severe is your LBP on a scale of 1 to 10, with 10 being the most severe?	
1-3	158 (26.2)
4-6	160 (26.5)
7-10	44 (7.3)
No pain	242 (40.1)
Was this pain bad enough to limit your regular activities or change your daily routine for >1 day?	
Yes	131 (21.7)
No	473 (78.3)
Years of working in the office?	
<5 years	191 (31.6)
5-10 years	256 (42.4)
>10 years	157 (26)
How many hours per day do you spend sitting at your desk?	
<8 hours	70 (11.6)
8 hours	388 (64.2)
>8 hours	146 (24.2)
Weekly rest period	
None	13 (2.2)
1 day	78 (12.9)
2 days	472 (78.1)
>2 days	41 (6.8)
Have you made any changes to your workstation or work habits to reduce your risk of LBP?	
Yes	281 (46.5)
No	323 (53.5)
Satisfaction with job	
Yes	472 (78.1)
No	132 (21.9)
Work stress in the past month?	
Always	126 (20.9)
Frequently	139 (23)
Sometimes	228 (37.7)
Rarely	71 (11.8)
Never	40 (6.6)

**Figure 2:**
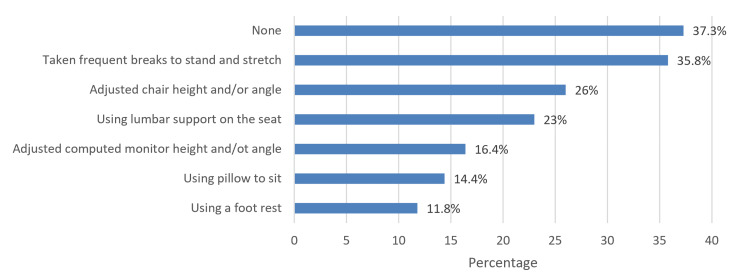
Specific changes made in a workstation or work habits to reduce the risk of LBP. LBP, low back pain

In Figure 2, of those who made changes to workstations/work habits to reduce the risk of LBP (*n *= 281), the most common changes done in a workstation/work habit to reduce the risk of LBP was taking frequent breaks to stand or stretch at work (216, 35.8%) followed by adjusted chair height and/or angle (157, 26%).

When assessing the relationship between LBP and sociodemographic and clinical variables (Table 3), it became evident that the prevalence of LBP was significantly higher among females (*P* = 0.013), individuals who were overweight or obese (*P* = 0.001), those who did not engage in physical exercise (*P* < 0.001), individuals experiencing weekly sleep disturbances (*P* < 0.001), those with a history of previous back trauma (*P* < 0.001), those with increasing years of working in the office (*P* = 0.034), individuals who made changes to their workstation or work habits to reduce the risk of LBP (*P* < 0.001), those who reported job dissatisfaction (*P* < 0.001), and those who frequently experienced work-related stress (*P* < 0.001).

**Table 3:** Relationship between LBP among the sociodemographic and clinical characteristics of participants (n = 604). ^§^*P*-value has been calculated using the chi-square test. ^**^Significant at *P *< 0.05 level. LBP, low back pain

Factor	LBP		*P*-value^§^
	With LBP (n = 362), *n* (%)	Without LBP (*n *= 242), *n* (%)	
Age group (years)			
18-30	154 (42.5)	109 (45)	0.139
31-40	108 (29.8)	83 (34.3)	
>40	100 (27.6)	50 (20.7)	
Gender			
Male	172 (47.5)	140 (57.9)	0.013^**^
Female	190 (52.5)	102 (42.1)	
Smoker			
Yes	84 (23.2)	51 (21.1)	0.538
No	278 (76.8)	191 (78.9)	
BMI level			
Normal or underweight	163 (45)	141 (58.3)	0.001^**^
Overweight or obese	199 (55)	101 (41.7)	
Physical exercise per week			
Yes	198 (54.7)	171 (70.7)	<0.001 **
No	164 (45.3)	71 (29.3)	
Sleep disturbance per week			
Yes	268 (74)	92 (38)	<0.001^**^
No	94 (26)	150 (62)	
Previous history of back trauma			
Yes	104 (28.7)	13 (5.4)	<0.001^**^
No	258 (71.3)	229 (94.6)	
Years of working in the office			
<5	124 (34.3)	67 (27.7)	0.034^**^
5-10	138 (38.1)	118 (48.8)	
>10	100 (27.6)	57 (23.6)	
Number of hours spent sitting on the desk			
<8	36 (9.9)	34 (14)	0.296
8	238 (65.7)	150 (62)	
>8	88 (24.3)	58 (24)	
Have you made any changes to your workstation or work habits to reduce your risk of LBP?			
Yes	213 (58.8)	68 (28.1)	<0.001^**^
No	149 (41.2)	174 (71.9)	
Satisfaction with job			
Yes	263 (72.7)	209 (86.4)	<0.001^**^
No	99 (27.3)	33 (13.6)	
Work stress in the past month			
Always/Frequently	182 (50.3)	83 (34.3)	<0.001^**^
Sometimes	132 (36.5)	96 (39.7)	
Rarely/Never	48 (13.3)	63 (26)	

In a multivariate regression model (Table 4), it was observed that the independent risk factors for LBP include being overweight or obese, having sleep disturbance per week, having a previous history of back trauma, increasing years of working in the office, changing workstation/work habits to reduce the risk of LBP, and always/frequently experience work stress while the protective factors for LBP include physical exercise per week and satisfaction with the job. This further indicates that compared to office workers who were normal or underweight, the risk for LBP among office workers who were overweight or obese was likely to increase by at least 1.74 times higher (adjusted odds ratio [AOR] = 1.737; 95% CI = 1.143-2.640; *P *= 0.001). Office workers who experienced sleep disturbance per week were 3.52 times more likely to be associated with the risk of LBP compared to office workers who did not experience sleep disturbance (AOR = 3.519; 95% CI = 2.318-5.344; *P *< 0.001). Office workers who had a previous history of back trauma were predicted to increase the risk of having LBP by at least 8.9-fold higher (AOR = 8.906; 95% CI = 4.440-17.865; *P *< 0.001). Respondents who were working for more than 10 years in the office were predicted to increase the risk of LBP by at least 2.4 times higher compared to those who were working for less than five years (AOR = 2.377; 95% CI = 1.402-4.030; *P *= 0.011). Respondents who made changes to workstations/work habits to reduce the risk of LBP were 4.3 times higher to be more associated with the risk of LBP (AOR = 4.289; 95% CI = 2.741-6.712; *P *< 0.001). Also, respondents who always/frequently experience work stress were 2.63 times more likely to be associated with the risk of LBP than those who rarely/never experienced work stress (AOR = 2.630; 95% CI = 1.498-4.616; *P *= 0.001). In contrast, respondents who were doing regular exercise were predicted to decrease the risk of LBP by at least 52% (AOR = 0.487; 95% CI = 0.315-0.751; *P *= 0.001), and those who were satisfied with their job were also predicted to decrease the risk of LBP by at least 67% (AOR = 0.338; 95% CI = 0.192-0.595; *P *= 0.001).

**Table 4:** Multivariate regression analysis to determine the significant independent predictor associated with LBP (n = 604). ^**^Significant at *P *< 0.05 level. AOR, adjusted odds ratio; CI, confidence Interval; Ref, reference category

Factor	AOR	95% CI	*P*-value
Gender			
Male	Ref		
Female	1.464	0.975-2.199	0.066
BMI level			
Normal or underweight	Ref		
Overweight or obese	1.737	1.143-2.640	0.010^**^
Physical exercise per week			
Yes	0.487	0.315-0.751	0.001^**^
No	Ref		
Sleep disturbance per week			
Yes	3.519	2.318-5.344	<0.001^**^
No	Ref		
Previous history of back trauma			
Yes	8.906	4.440-17.865	<0.001^**^
No	Ref		
Years of working in the office			
<5	Ref		
5-10	0.947	0.548-1.636	0.845
>10	2.377	1.402-4.030	0.001^**^
Have you made any changes to your workstation or work habits to reduce your risk of low back pain?			
Yes	4.289	2.741-6.712	<0.001^**^
No	Ref		
Job satisfaction			
Yes	0.338	0.192-0.595	0.001^**^
No	Ref		
Work stress in the past month			
Always/Frequently	2.630	1.498-4.616	0.001^**^
Sometimes	1.572	0.970-2.548	0.066
Rarely/Never	Ref		

## Discussion

This study seeks to determine the prevalence and risk factors for LBP among office workers from different regions (central, eastern, western, southern, and northern) of Saudi Arabia. Several papers discussed LBP prevalence in various professions, but none tackled the pain dilemma experienced by office workers. Thus, this study shed some light on their predicament. The results of this study showed that the overall office workers' LBP prevalence was 59.9%. This prevalence is higher than the LBP prevalence of office workers in Lebanon [2] and among Taif nurses [14], with 44.8% and 48.4%, respectively. However, in a systematic review conducted by Aldera et al. [13], the range of LBP in various professional groups and within working age groups varies between 64% and 89%, which was higher than our report. Notwithstanding these reports, several papers documented a lower point prevalence of LBP, but the lifetime prevalence was higher, ranging from 61.6% to 92.1% [1,8,12]. LBP is a common problem among office workers. We emphasized the importance of support and measures from the management to reduce the impact of predisposing risk factors of LBP.

According to the study by Bawab et al. [2], females were affected more by the LBP than males. This has been supported by Shiri et al. [10], LBP and lumbar radicular pain were more prevalent in women than men. Our office workers echoed this scenario, wherein significantly more female respondents experienced LBP than male respondents. Based on previous reports, females with a high physical workload had a 78% increased risk of developing LBP than those with a low physical workload [16]. Hence, an implication for a more relaxing environment should focus on women prone to developing LBP.

Being overweight or obese is susceptible to various comorbidities, including LBP. This has been reflected in our results. Data in our study suggest that office workers classified as overweight or obese were at increased risk for developing LBP by at least 1.8 times higher than those with lower BMI levels (<25 kg/m^2^). Consistent with our findings, Spyropoulos et al. [1], as well as Alnaami et al. [15] found significant associations between increased BMI and the risk of LBP.

History of back trauma increased the risk of LBP by at least 8.9 times higher than those without back trauma history. This mirrored the results of Rezaee et al. [12], wherein the history of LBP increased the likelihood of occurrence of LBP. Interestingly, Bawab et al. [2] disclosed that LBP was positively associated with backbone crookedness, numbness, wrist pain, contractions, knee pain, and previous treatment for back pain, while Alnaami et al. [15] identified a positive history of overexertion back trauma as a risk factor for LBP.

Psychological disorders could also lead to LBP. Based on our regression model, increasing work stress was directly correlated with an increased risk of developing LBP. Another significant risk factor for LBP is sleep disturbance, which increases the risk for LBP by at least 3.5-fold. Also, increasing years of office work was associated with an increased risk for LBP. In contrast, age group, smoking status, and number of hours spent sitting at the desk were found to be nonrelevant factors for LBP (*P *> 0.05). In southwestern Nigeria [9], LBP was significantly associated with senior staff grade and smoking, while the severity of LBP greatly influences sitting for more than three hours. However, in Malaysia [7], they found no significant association between LBP and all demographic variables involved, such as age, gender, level of education, ethnicity, smoking, work status, years of working, type of chair, attended an ergonomic course, duration of siting, duration of each leaving (minutes), and frequency of leaving the chair (*P *> 0.05), which was similarly detected in Taif study [14].

Conversely, despite the identified significant risk factors, we also discovered factors that could reduce LBP. Our results suggest that engagement in physical exercise and satisfaction with a job might be protective factors for developing LBP, wherein performing physical exercise per week could decrease the risk for LBP by at least 50%. In contrast, the likelihood effect could decrease by 65% for those who were satisfied with their job. Job satisfaction was also identified as a protective factor for LBP along with ergonomic chairs and doctor consultation and undergoing radiography, as reported by Bawab et al. [2], while in a study by Shiri et al. [10], as well as Alnaami et al. [15], both studies documented physical exercise as a factor that alleviates the risk for LBP.

## Conclusions

There was a high prevalence of LBP among office workers in Saudi Arabia. Elevated BMI, sleep disturbance per week, previous history of back trauma, increasing years of working in the office, changing workstations or habits to reduce the risk of LBP, and experiencing work stress in the past months were identified as the independent risk factors for LBP while performing physical exercise per week and being satisfied with a job was recognized as the protective factors for LBP. This study provides evidence that LBP greatly impacts office workers' working conditions. We emphasized the importance of a healthy lifestyle, ergonomic balance and control, good posture, and cultivating educational programs to prevent LBP among office workers.
